# Lysine-Specific Demethylase 1 (LSD1)-Mediated Epigenetic Modification of Immunogenicity and Immunomodulatory Effects in Breast Cancers

**DOI:** 10.3390/curroncol30020164

**Published:** 2023-02-09

**Authors:** Dong Yeul Lee, Talha Salahuddin, Jabed Iqbal

**Affiliations:** 1 Department of Anatomical Pathology, Singapore General Hospital, 20 College Road, Academia, Level 10, Diagnostics Tower, Singapore 169856, Singapore; 2 School of Biological Sciences, Nanyang Technological University, 60 Nanyang Drive, Singapore 637551, Singapore; 3 Faculty of Medicine, Dentistry and Health Sciences, The University of Melbourne, Grattan Street, Parkville, VIC 3010, Australia

**Keywords:** breast cancer, epigenetics, methylation, lysine-specific demethylase 1, LSD1, tumor immunogenicity, immune-checkpoint, immunotherapy

## Abstract

Tumor evolution to evade immune surveillance is a hallmark of carcinogenesis, and the modulation of tumor immunogenicity has been a challenge to present therapeutic responses in immunotherapies alone for numerous cancers. By altering the cell phenotype and reshaping the tumor microenvironment, epigenetic modifications enable tumor cells to overcome immune surveillance as a mechanism of cancer progression and immunotherapy resistance. Demethylase enzymatic activity of lysine-specific demethylase 1 (LSD1), a histone demethylase first identified in 2004, plays a pivotal role in the vast cellular processes of cancer. While FDA-approved indications for epigenetic therapies are limited to hematological malignancies, it is imperative to understand how epigenetic machinery can be targeted to prime immunotherapy responses in breast cancers. In this review, we discuss the potential roles of epigenetics and demethylating agent LSD1 as a potent new cancer management strategy to combat the current challenges of breast cancers, which have presented modest efficacy to immune checkpoint inhibitors till date. Additionally, we describe the combined use of LSD1-specific inhibitors and immune checkpoint inhibitors in existing breast cancer preclinical and clinical trials that elicits a robust immune response and benefit. Overall, the promising results observed in LSD1-targeting therapies signify the central role of epigenetics as a potential novel strategy to overcome resistance commonly seen in immunotherapies.

## 1. Introduction

Over the past decade, advancements in immunotherapy as an anticancer therapy have revolutionized patient responsiveness to treatment. To date, immune-checkpoint blockade (ICB) therapies targeting cytotoxic T lymphocyte-associated antigen 4 (CTLA4/CD152), programmed cell death protein 1 (PD-1/CD279), and programmed death-ligand 1 (PD-L1) have presented unprecedented responses in significant percentages of cancer patients [1]. Even then, efficacy and response rates vary according to cancer types and particular ICB regimens. Researchers have therefore since attempted to find ways to optimize immunotherapy and overcome immune checkpoint inhibitor resistance. Of note, only a minority of breast cancer patients clinically benefited from ICBs, with a relatively low overall response rate [2]. 

It is now established that epigenetic dysregulation can be involved in the pathogenesis and development of cancers. Defects in chromatin modifiers have been described in hematological and solid malignancies, where there has been increasing evidence of a correlation between the role of aberrant epigenetics and cancer etiology [3,4,5]. More importantly, the reconfiguration of immune cell chromatin landscapes in conjunction with the extensive modification of tumor cell epigenome can modulate and enhance antitumor immunity or immunotherapy responses to improve overall disease outcomes [6]. In the promising field of new drug discovery in epigenetic enzyme-targeted therapy, extensive research works to date have also demonstrated that histone deacetylases (HDAC) and DNA methyltransferases (DNMT) are druggable targets in cancer therapeutics. Nevertheless, active clinical trial investigations on histone methyltransferases and lysine demethylase inhibitors are being evaluated as the latest potential group of epi-drugs [7]. 

Thus, this review aims to evaluate the potential of targeting epigenetic modifier lysine-specific demethylase 1 (LSD1) in offering a novel direction to overcome limitations seen in immunotherapies. We focus on the underlying basis of antitumor immunity and how targeting epigenetic marker LSD1 can provide an avenue to modulate tumor immunogenicity and the abysmal immunotherapy response rate seen in breast cancers. 

## 2. Aberrant Epigenetic Modification Patterns in Tumorigenesis

**Figure 1:**
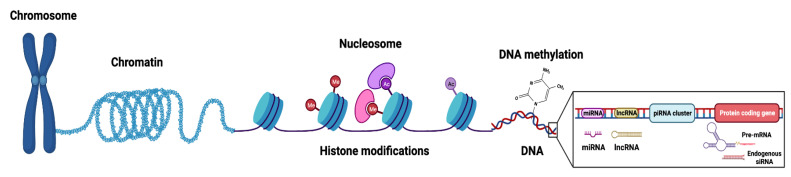
Graphical representation of chromatin structure and primary epigenetic mechanisms (histone modifications, DNA modifications, and non-coding RNA regulation) that regulate gene expression. The nucleosome, composed of DNA wrapped around an octamer core of histone proteins, is the functional unit of chromatin. Histone modifications involve numerous post-translational modifications, mainly targeting amino acid residues of *N*-terminal tails of the histones. DNA modifications refer to covalent modifications of the DNA at position 5′ of cytosine in CpG dinucleotides and are mainly facilitated by a family of enzymes known as DNA methyltransferases (DNMTs) or ten-eleven translocation (TETs) demethylases. Non-coding RNAs (ncRNAs), which do not encode proteins, can be subcategorized based on their size into long (lncRNAs, >200 nucleotides) and small (sncRNA, <200 nucleotides) non-coding RNAs with various regulatory roles in epigenetics (such as chromatin remodeling, gene transcription, mRNA degradation). sncRNAs include small inhibiting RNAs (siRNAs), microRNAs (miRNAs), and PIWI-interacting RNAs (piRNAs). Figure created with BioRender.com (accessed on 26 January 2023).

**Figure 2:**
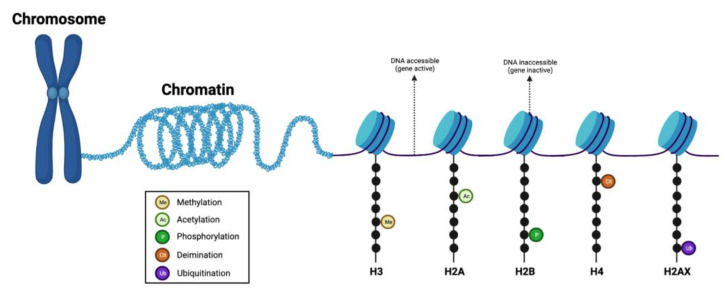
Graphical representation of the histone complex and modifications. Histone modifications determine the extent of chromatin wrapping around the histone proteins. Protruding histone tails from the nucleosome can be modified by various post-translational modifications (such as methylation, acetylation, phosphorylation, ubiquitination, and deamination) at different residues. Loosely-coiled chromatin contains transcriptionally accessible DNA regions, while tightly-coiled chromatin contains transcriptionally inaccessible DNA regions. Figure created with BioRender.com (accessed on 26 January 2023).

The primary mechanisms of epigenetic modifications include histone modifications, DNA methylation, nucleosome accessibility, and regulatory non-coding RNAs (miRNAs, piRNAs, endogenous siRNAs, long non-coding RNAs/lncRNAs) (Figure 1) [8,9]. The histone complex, composed of two unstable dimers (H2A, H2B) and tetramers (of H3 and H4), facilitates genomic DNA condensation and impacts post-translational modifications on the conserved lysine residues of histone tails. Such post-translational modifications include acetylation, methylation, ubiquitination, phosphorylation, sumoylation, and deamination (Figure 2) [10]. Histone methylation, which often occurs at lysine (K) residues of histone H3 and H4, is facilitated by enzyme histone methyltransferases to transfer methyl groups onto the lysine residues—acting as active or repressive marks of gene expression [11]. 

**Figure 3:**
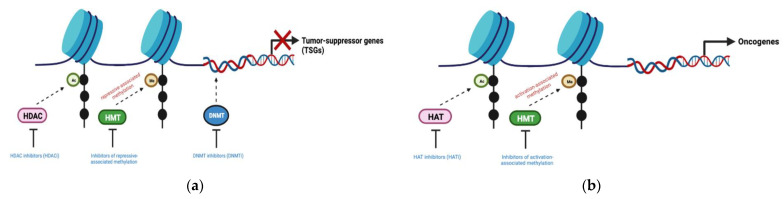
Aberrant epigenetic mechanisms in cancer and the specific application of epigenetic modifier inhibitors to reverse its effects. (**a**) Tumor suppressor silencing observed in cancers can be induced by DNA methylation (with DNMTs), histone deacetylation (with HDACs), or repressive histone methylation (with HMTs); (**b**) Oncogene activation observed in cancers can be induced by activation-associated histone hypermethylation (with HMTs) or histone hyperacetylation (with HATs). DNMT (DNA methyltransferase); HMT (Histone methyltransferase); HDAC (Histone deacetylase); HAT (Histone acetyltransferase). Figure created with BioRender.com (accessed on 30 December 2022).

Tumor suppressor-mediated gene silencing and oncogene activation are hallmarks of aberrant epigenetics, a common feature seen in cancers. This is characterized by global DNA hypomethylation as well as accompanying hypermethylation at region-specific sites of CpG islands near gene-regulatory regions, such as tumor-suppressor genes (TSGs) [12]. DNA methylation within particular gene segments may also drive methylation-related mutational events within cells, contributing to cancer development and increased genetic diversity of tumors [13]. While induced effects of DNA methylation and histone modifications are linked with epigenetic dysregulation, the epigenetic modulation process can be reversed with epigenetic modifier inhibitors (Figure 3). Furthermore, epigenetic modulators such as DNA methyltransferase inhibitors (DNMTis) and histone deacetylase inhibitors (HDACis) can re-program the tumor immune microenvironment to increase the susceptibility of tumor cells to T-cell mediated cytotoxicity, thereby leading to enhanced anti-tumor immune responses. 

## 3. Mechanisms of Immune-Checkpoint Blockade (ICB) Resistance and Cancer Epigenetics

### 3.1. Immune-Checkpoint Blockade (ICB) Therapies

Many decades ago in 1968, reactivity seen in isolated lymphocytes from cancer patients against cancer cells gave rise to the potential of cancer immunotherapy [14]. While chemotherapy, radiotherapy, and surgery have long been considered the basis of cancer treatment, the first successful FDA-approved ICB drug in 2011—the anti-CTLA-4 monoclonal antibody ipilimumab [15]—revolutionized immunotherapy as a new pillar of cancer therapy. Contrary to traditional cytotoxic therapies, ICB drugs function to augment durable host immune responses with less toxicity. 

Inhibitory immune checkpoints that are well-described include CTLA-4, PD-1, and PD-L1 [16,17]. CTLA-4 molecule is overexpressed on the active T cell surface, preventing excessive T cell receptor (TCR) stimulation via competitive binding with CD28 co-stimulatory receptor, to bind against its ligands (B7-1/CD80, B7-2/CD86). Similarly, PD-1 is upregulated on activated T cells and binds to its PD-L1 ligand—limiting T cell activation. Overall, a specific blockade on the aforementioned molecules sustains anti-tumor responses [16,18]. Years later, other ICB agents targeting PD-1 (nivolumab, pembrozulimab, cemiplimab, dostarlimab) and PD-L1 (atezolizumab, avelumab, durvalumab) were discovered and approved for clinical use against solid malignancies [19,20]. While continual clinical development of ICBs and understanding of tumor immunology show great promise, only a small percentage of patients achieve a response to monotherapy. Hence, further research efforts to optimize immunotherapy options and exploration of new molecules in the application of ICB therapy are required. In particular, the identification of established factors that can influence patient treatment outcomes is necessary as well. One such mechanism is epigenetic remodeling, which is involved and essential in reprogramming enhanced antitumor immune response. Several recent studies have demonstrated that epigenetic modifiers (SETDB1, LSD1) can regulate tumor cell-intrinsic immunity and T-cell exhaustion [21,22,23]—shedding new light on leveraging the potential of epitherapy to specifically improve the effectiveness of immunotherapies.

### 3.2. Tumor Resistance to Immune Checkpoint Inhibition

A major challenge in ICB therapy is overcoming immune checkpoint inhibitor (ICI) tumor resistance [24]. Clinical progression on ICIs is broadly categorized into (i) primary resistance (irresponsive to checkpoint inhibition), (ii) adaptive resistance (functional antitumor response limited by immunosuppression), and (iii) acquired resistance (initial response followed by eventual disease progression or relapse) [25,26]. 

Primary and adaptive resistance to immunotherapy can be attributed to both tumor cell-intrinsic and/or tumor cell-extrinsic factors. Multiple tumor-intrinsic mechanisms that lead to primary and/or adaptive resistance include the lack of T cell responses due to loss of tumor antigen recognition/expression/presentation, as well as the expression and repression of certain genes/pathways within tumor cells that limit the immune function within the tumor microenvironment (TME) [26]. The presence of such mechanisms could exist at the time of initial presentation (termed “primary resistance mechanisms”) or may potentially evolve later (termed “adaptive resistance mechanisms”). Meanwhile, tumor cell-extrinsic mechanisms that contribute to the inhibition of antitumor immune responses involve non-tumor cell components within the microenvironment such as regulatory T cells (Tregs), myeloid-derived suppressor cells (MDSCs), M2 macrophages, and other inhibitory immune checkpoints [26]. Other potential mechanisms of disease relapse and progression upon the brief therapeutic response seen in certain patients include the loss of T cell functional phenotype, downregulation of tumor antigen presentation, and development of escape mutation variants [26]. 

In the PD-1 checkpoint pathway, the PD-1 inhibitory receptor has roles in T cell dysfunction during cancer development where studies have proven PD-1 expression in exhausted T cells is driven by demethylation of the PD-1 promoter [27]. The degree of epigenetic program stability can limit the maintenance of long-term effector function and memory development by T cells following PD-1 blockade [28], and thus present a potential explanatory role of epigenetic fate inflexibility on refractory disease observed in anti-PD-1 and/or anti-PD-L1 treated patients. More importantly, selective targeting in a subset of exhausted CD8 T cells may sufficiently enhance sustained effector function and durable antitumor responses in future clinical studies [29].

### 3.3. T Cell Dysfunction

T cell dysfunction refers to the cytotoxic T cells within the TME that have turned ineffective or immunotolerant, thereby conferring both primary and acquired resistance. Prolonged signaling to T cell receptors, due to persistent antigen exposure, increases the expression of inhibitory immune checkpoint receptors, which in turn drives “T cell dysfunction” [30]. Chronic antigen stimuli also influence the increased level of PD-1 expression through the NFAT cytoplasmic 1 (NFATc1)-mediated pathway, which is involved in the maintenance of exhausted phenotype [31,32]. Moreover, simultaneous expression of inhibitory co-receptors (such as PD-1, CTLA-4, TIM-3) is correlated with increased T cell dysfunction in cancer and disease progression [33]. With an increasing proportion of T cells co-expressing such receptors or on tumor-infiltrating lymphocytes (TILs), the functionality of T cells decreases and ultimately leads to tumor progression [33]. 

### 3.4. T-Cell Exhaustion

Characterized by sustained upregulation of multiple checkpoint proteins (PD-1, TIM-3, CTLA-4, LAG-3), exhausted T cells are a distinct group of dysfunctional T cells with poor effector function that arise in response to chronic viral infections and cancer [30]. This process is driven by persistent antigen exposure in the TME, alongside other early events that initiate T cell activation, which is critical for the reprogramming of exhaustion in the tumor [34]. In addition, Pauken et al. [28] found out that long-term blockade of the PD-1 pathway eventually led to T cell “re-exhaustion”, as well as observed altered transcriptional programs in such exhausted T cells [35,36]. Furthermore, epigenetic changes (DNA methylation, histone modifications) are essential processes known to drive the differentiation of T cells, and therefore, exhausted T cells are often found to have altered epigenomes compared to normal functioning T cells [37]. As such, elevated lysine-specific demethylase 1 (LSD1) levels are reported to be a major contributor to the exhausted T cell phenotype [38], where targeting LSD1 in exhausted T cells of immunotherapy-resistant mice noticed increased T cell effector functionality (corresponding to elevated IFN levels and greater T cell infiltration). In the following sections, this review will explore LSD1 and its essential role in the tumor immune response and microenvironment. 

## 4. Lysine-Specific Demethylase 1 (LSD1/KDM1A)

**Figure 4:**
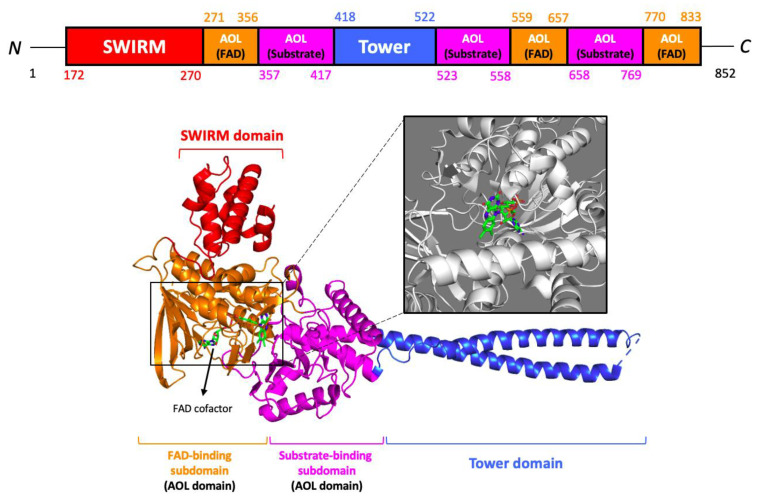
Ribbon structure of lysine-specific demethylase 1 (LSD1). The SWIRM domain is colored red, the AOL domain is colored orange (FAD-binding subdomain) and purple (substrate-binding subdomain), and the Tower domain is colored blue. FAD cofactor is displayed as a stick model. Data on the X-ray diffraction structure were obtained from Protein Data Bank (PDB) [accession no. 2HKO]. Figures were prepared using the programs of PyMOL molecular visualization systems.

The 852-residue sequence of LSD1 contains three main protein domains: the *N*-terminal SWIRM (Swi3p/Rsc8p/Moira) domain, a central protruding Tower domain, and a *C*-terminal AOL (amine oxidase-like) domain with two well-defined subdomains—the flavin adenosine dinucleotide (FAD) cofactor-binding subdomain and a substrate-binding subdomain [39,40] (Figure 4). Interestingly, LSD1 is found to be highly conserved in different organisms (both unicellular and multicellular eukaryotes), ranging from yeast Schizosaccharomyces pombe to humans [41,42]. The evolutionarily conserved SWIRM domain is found in many chromosomal proteins involved in chromatin modifications or remodeling. Recent reports on the structures of SWIRM domains were reported for mouse Ada2α (transcriptional adaptor-2), yeast Swi3 (switching deficient-3), and human LSD1 [43,44,45]. Revelations on the SWIRM domain structures, whereby the LSD1 SWIRM domain consists of a helical bundle containing a long central alpha helix that is surrounded by several shorter helix motifs, implicate a conserved SWIRM domain fold [46]. Additionally, close interactions between the SWIRM domain and amine oxidase domain through an extensive hydrophobic interface, forming a highly conserved cleft in the vicinity of the active site, may serve as an additional histone tail-binding site. The Tower domain, inserted into the AOL domain with a long helix-turn-helix structure, comprises binding spots for LSD1-interacting proteins such as CoREST (co-repressor for RE-1 silencing transcription factor), CtBP1 (carboxyl-terminal binding protein 1), HDAC1/2, and Snai1. The corepressor CoREST-binding domain is indispensable for the histone demethylase activity of LSD1, where a deletion mutant (LSD1ΔTower) replaced by a pentaglycine loop elicits an inability to reduce methylation of H3K4 [47]. 

Initially identified as a transcriptional repressor, LSD1 is also a component of the Mi-2/nucleosomal remodeling deacetylase (NuRD) complex with roles in influencing the repressive chromatin state and DNA damage repair processes [48]. While LSD1 generally demethylates monomethyl and dimethyl-histone H3K4 (H3K4me1/2) methylation marks, LSD1 also acts as a co-activator in androgen (AR) and estrogen (ER) receptor-dependent transcription through demethylation of repression-associated monomethyl and dimethyl-histone H3K9 (H3K9me1/2) marks upon hormone binding [49,50]. 

**Figure 5:**
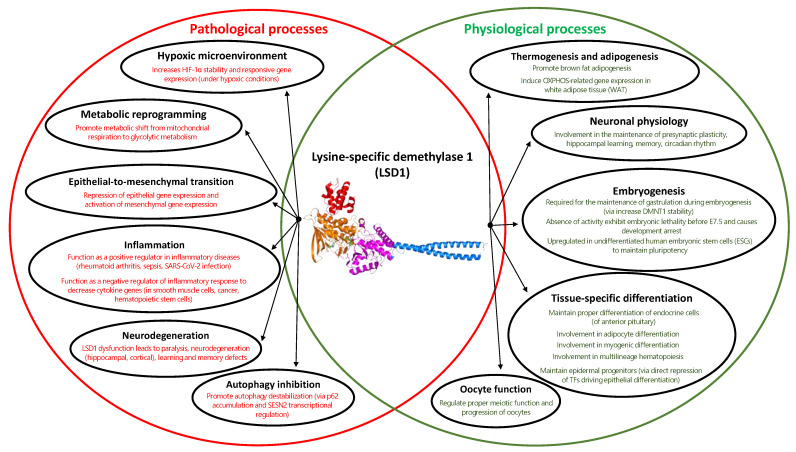
Venn diagram on the comprehensive roles of LSD1 function in both physiological (colored in green) and pathological (colored in red) conditions.

Pleiotrophic roles of LSD1 regulation in several cellular processes suggest its association with normal physiological and pathological processes [51,52,53,54] (Figure 5). To name a few, LSD1’s involvement in the stabilization of HIF-1α under hypoxic conditions [51], metabolic shift towards glycolysis [55], epithelial-to-mesenchymal transition (EMT) [56,57,58], inflammatory responses [51], defective autophagy [59,60], and its dysfunction contributing to the development of neurodegenerative diseases [61,62,63], underlies its association with several diseases. On the other hand, LSD1 is critical for adipogenesis and thermogenic gene regulation [64,65,66,67], regulation of gene expression in various neuronal physiology (circadian clock, presynaptic plasticity, hippocampal learning, and memory) [68,69], maintenance of embryonic development or pluripotency [70,71,72,73], the proper meiotic function of oocytes [74], and tissue-specific differentiation (endocrine cells of anterior pituitary [72], skeletal muscles [75], adipocytes [76], multilineage hematopoeisis [77,78], maintenance of epidermal progenitors [79]). With a broad spectrum of cellular processes under the regulation of LSD1, dysregulated protein expression can greatly increase cancer risk and is observed in multiple aggressive cancers [80]. Taken together, there is increasing evidence to suggest LSD1 as an epigenetic master regulator that controls cellular homeostasis and can be a potential therapeutic target in overcoming several diseases.

## 5. LSD1 and Tumor Immunogenicity

Blankenstein et al. [81] defined tumor immunogenicity as the ability of tumors to induce an immune response that can prevent their growth. Fundamental determinants of tumor immunogenicity, namely, include tumor antigenicity, antigen processing, and the efficacy of antigen presentation [82]. While there is extensive ongoing research investigating genomic features underlying tumor immunogenicity mechanisms and its biomarkers, the role of methylation as a biomarker for tumor immunogenicity remains an area that is not well established [83,84]. 

### 5.1. Methylation Status and Tumor Immunogenicity

DNA methylation modulates chromatin remodeling and RNA transcription, influencing cancer at the cellular level. Aberrant methylation status (promoter hypermethylation and hypomethylation) has been reported to be involved in tumorigenesis across numerous cancer types [85,86]. CpG islands within the human genome are mainly located in gene promoters, where promoter hypermethylation in tumor-suppressor genes is involved in downstream gene silencing to promote carcinogenesis [87]. This is further supported by Costello et al.’s [88] study on 1200 unselected CpG islands from 98 primary cancers, whose results found hypermethylation to be much more common in the cancer genome compared to that of normal control cell samples. Recent studies further prove that inhibiting DNA methylation alone or coupled with HDAC inhibitors can activate the tumor interferon (IFN) pathway and increase cancer immunotherapy responses [89,90]. 

### 5.2. Potential Regulatory Role of LSD1 in Breast Cancer Immunogenicity

**Figure 6:**
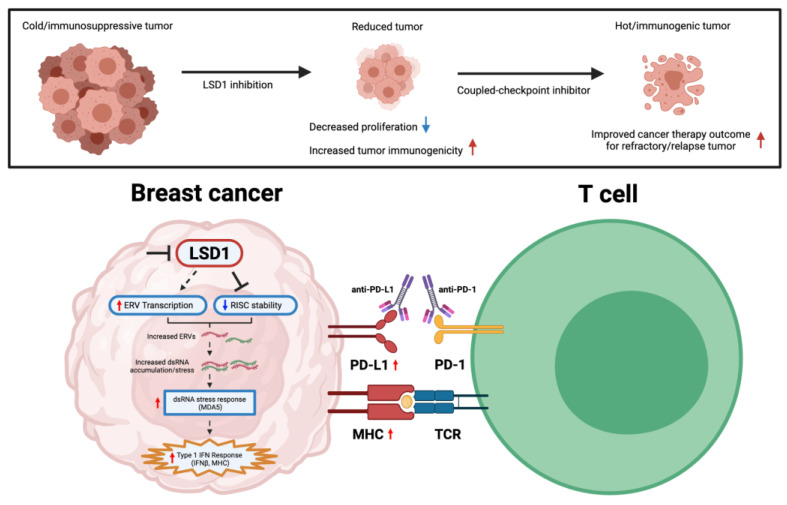
Graphical representation of mechanistic effect upon LSD1 inhibition in eliciting tumor immunogenicity in breast cancers. Red arrows indicate increased level, while blue arrows indicate reduced level. Figure created with BioRender.com (accessed on 2 January 2023).

The discovery and significant success of ICB introduction largely increased patient survival in a subgroup of tumors (melanoma, non-small cell lung carcinoma, renal cell carcinoma, lymphoma, head and neck squamous cell carcinoma); even then, the clinical response varied depending on tumor immunogenicity and degree of lymphocyte infiltration into tumor stroma [91]. In addition, breast cancers, unlike most immunotherapy-responsive tumors, generally harbor lower tumor mutational burden (TMB) and lymphocyte infiltration—both of which are characterizations of reduced immunogenicity and the “cold” immunogenic nature of the disease. As a result, immunotherapy response in breast cancer patients remains modest. ICB benefit in breast cancer was first observed in 2018 for the metastatic/unresectable triple-negative breast cancer (TNBC) IMpassion130 trial, which studied the use of atezolizumab (anti-PD-L1 inhibitor) combined with nab-paclitaxel. However, the atezolizumab benefit was only restricted to a subset of the 41% PD-L1 positive TNBC population (that expressed PD-L1 in >1% cells) with marginally improved survival (absolute median benefit of 2.5 months) [91,92,93]. Given the limited efficacy seen in ICI monotherapy in breast cancers to date, recently extended interest in combining with other therapeutic modalities has arisen. For instance, epitherapy possesses the capacity of leveraging the epigenetic landscape in the TME that is supportive of long-term immunotherapy responses. As alluded to earlier, Pauken et al. [28] concluded that due to the negligible effect of PD-1 blockade on the epigenetic landscape of exhausted T cells, combining epigenetic-modifying drugs with ICB could potentially improve T cell reinvigoration. To date, several promising results of enhanced tumor immunogenicity upon LSD1-ablation in breast cancers are summarized in Table 1 and Figure 6. 

While numerous studies have demonstrated DNMT inhibitors to promote type I IFN activation and increase tumor response to anti-CTLA therapeutics through cytosolic antiviral double-stranded RNA (dsRNA)-sensing pathways, the full spectrum on how chromatin regulators can modulate tumor immunity and immunotherapy in breast cancer is still poorly understood. Of note, Sheng et al. [94] demonstrated that LSD1 inhibition in human cancer cells stimulates dsRNA stress and IFN activation, subsequently sensitizing tumors to T cell immunity and infiltration. 

In the TNBC subtype, Qin et al. [95] presented inversely correlated data between LSD1 with key cytotoxic T cell-attracting chemokines (CCL5, CXCL9, CXCL10) and PD-L1. This is further supported by combinatorial PD-1 mAb coupled LSD1-inhibition therapy in exhibiting superior reduced tumor progression/tumor metastasis, reduced CD4+/CD8+ T cell ratio, and increased nodal CD3+/CD8+ T cell populations. The overall reduction in the CD4+/CD8+ ratio of TILs is a suggestive indicator of enhanced antitumor immunogenicity capacity [96]. Consistently, oral administration of INCB059872 (FAD-directed covalent inhibitor of LSD1) in the 4T1 mammary cancer model significantly inhibits polymorphonuclear (PMN)-MDSC differentiation and increased intratumoral T lymphocyte infiltration [97]. The combination of both INCB059872 and anti-PD-L1 treatment further enhanced antitumor efficacy, restoring the responsiveness to PD-1/PD-L1 axis blockade. Additionally, tumor-initiating cells (T-ICs; also known as cancer stem cells/CSCs) are specific subsets of cells with a high propensity for multidrug resistance and tumor self-renewal. Due to the lack of known therapeutic options in TNBC treatment, chemotherapy still remains a primary treatment regimen for patients; however, repeated exposure of tumors to chemotherapeutic drugs is a major cause of chemoresistance and metastatic potential—making T-ICs attractive antitumor targets [98]. There is increasingly compelling evidence to suggest epigenetic therapies can induce differentiation of “quiescent-state” T-ICs and improve therapeutic sensitization for drug-resistant and low-immunogenic tumors [99,100]. To overcome 5-fluorouracil (5-FU)-induced chemoresistant TNBC, the Ji et al. study [101] demonstrated that the codelivery of LSD1 inhibitor and 5-FU exhibited syngeneic effects in modulating the plasticity of TNBC T-ICs—including reduced Ki67 proliferation, suppressed tumor growth/volume/metastasis and increased CD8+ T cell infiltration. In line with prior studies, the combination therapy similarly observed induced potent T cell immunity via innate sensing of endogenous RNA stress. In particular, nuclear LSD1 phosphorylation at serine 111 (nLSD1p) is found to be enriched in both immunotherapy/chemoresistant-breast cancer cells with increased stem-like, mesenchymal signature [102]. More importantly, Tu et al. [38] demonstrated significant findings on the utilization of nuclear-axis targeting LSD1 inhibitors, which, compared to traditional FAD inhibitors, better inhibit CSCs and mesenchymal signature, enhance CD8+ T cell reinvigoration/transcriptional memory, and overexpress key-immune related pathways in therapy-resistant TNBCs. 

## 6. LSD1/KDM1A Demethylase Inhibitor in Breast Cancer Clinical Trials

### Current Status of LSD1 Inhibitor Use in Breast Cancer Clinical Trials

Given the importance of LSD1 enzymatic activity in cancer and its potential to enhance tumor immunogenicity, the development of specific and potent LSD1 pharmacologic inhibitors are encouraging. At present, there are many LSD1/KDM1A inhibitors under clinical trial as a promising future cancer therapy with the majority of these being targeted against small-cell lung cancer (SCLC) and acute myeloid leukemia (AML) [103,104]. The first LSD1 inhibitor drug to be identified was the nonselective monoamine oxidase inhibitor tranylcypromine (TCP), exerting inhibitory effects via covalent modification of its FAD cofactor to form an adduct in the binding pocket [53,105]. However, due to the relatively low potency of TCP against LSD1, other TCP derivatives and multiple compounds with irreversible/reversible LD1 inhibitory activity have been reported. To date, several irreversible and reversible LSD1 inhibitors including GSK2879552, IMG-7289 (Bomedemstat), INCB059872, ORY-1001 (Iadademstat/RG6016/RO7051790), ORY-2001 (Vafidemstat), pargylin, phenelzine, and tranylcypromine (TCP/PCPA)-based inhibitors, alone or in combination with other epi-inhibitors to target multiple modifications, have already shown synergistic antitumor effects in several malignancies [106,107,108,109]. 

Even then, evidence on the efficacy of single-agent LSD1-targeting compounds in pre-clinical studies for solid tumors is unwarranted. This is attributed to the multiple protein complexes that LSD1 associates with, where important structural roles may not be mediated through its enzymatic activity. For instance, treatment with TCP or TCP analogues in breast cancer cell lines inhibited proliferation, but at concentrations 20–30 fold higher than the half maximal inhibitory concentration (IC_50_) of LSD1. In addition, study findings on the use of inhibitors at substantially higher concentrations than that of the IC_50_ for LSD1 present a challenge to drawing solid conclusions.

With many of the known LSD1 inhibitors in early-phase trials for AML and SCLC, there are still comparably limited pre-clinical or clinical studies against solid malignancies including breast cancer. In this review, we probed into ClinicalTrials.gov (accessed on 8 January 2023), a registry of clinical trials run by the U.S. National Library of Medicine (https://clinicaltrials.gov/ (accessed on 8 January 2023)). The trials presented in this review are as of 8 January 2023, under the criteria that involved breast cancer (under “condition/disease” category) and LSD1 (under “others” category). Listed eligible trials are required to have published results to be evaluated in this review. Information on the trial identifier, trial title/acronym, trial result and side effects, patient cohort, and their breast cancer diagnosis are listed in Table 2. 

Of note, the use of phenelzine (an approved monoamine oxidase inhibitor/MAOi) in a preclinical study previously presented inhibitory effects against nuclear phosphorylated LSD1 with corresponding histone methylation and CSC inhibition [102]. Recently, the first-ever published phase I clinical trial on LSD1 inhibitor-coupled chemotherapy (phenelzine combined with nab-paclitaxel) in metastatic breast cancers was assessed to be safe with the potential for eliminating circulating tumor cells (CTC) with aggressive mesenchymal phenotype [110]. This result on the safe and feasible approach for combined LSD1 inhibition along with standard chemotherapy in the clinical setting with observed induced phenotypic change from an aggressive mesenchymal CTC to less-metastatic epithelial phenotype strongly suggests the importance of LSD1 inhibition in preventing metastasis and prolonging breast cancer patient survival as a therapeutic target. Further exploration in larger studies to support and investigate pharmacokinetics against specific nuclear LSD1 inhibitors is warranted. 

## 7. Conclusions

Over the last decades, epigenetic regulation of DNA-templated processes has been intensively studied. There is also a vast increase in study findings to suggest that epigenetic changes are key drivers of carcinogenesis, where epitherapy is a novel area of targeted therapy that aims to reverse some of these abnormalities accumulated in cancer cells. Evidence from both pre-clinical and ongoing clinical studies further suggests how epigenetic therapy, through differential signaling mechanisms involving the microenvironment, could prime ICB efficacy and induce T cell attraction. Overall, LSD1 is implicated to function as a negative regulator of antitumor immunity and immunotherapy responsiveness, where LSD1 inhibition potentially converts “cold” tumors (resistant to ICB blockade) to “hot” tumors (responsive to ICB blockade) in poorly immunogenic breast malignancies. It is also imperative for future studies to continually validate LSD1 functionalities on the cancer-associated epigenome and its potential modulation of tumor immunogenicity in poorly immunosuppressive tumors, where it could give rise to novel strategies for cancer management. To the best of our knowledge, this review is the first to comprehensively evaluate LSD1 inhibition effects on promising enhanced immunogenicity in breast cancer studies, alongside highlighting the limited clinical studies on LSD1-targeting therapies in breast cancer patients. Nonetheless, this information, along with the continual generation of promising clinical and pre-clinical results seen with epigenetic drugs against chromatin regulators such as LSD1 may signify the central role of epigenetics in breast cancer.

## Figures and Tables

**Table 1:** List of research studies conducted on LSD1 inhibition effects on tumor immunogenicity in breast cancers.

Study	LSD1 Inhibition Effects on Enhanced Tumor Immunogenicity in Breast Cancers
Sheng et al. [94]	LSD1 inhibitor (GSK-LSD1) induced upregulation of genes enriched for type I IFN response and antiviral response in MCF7 cells
	GSK-LSD1 upregulated IFN/antiviral responsive genes that induce IFNs and ISGs in MCF7 cells
	GSK-LSD1 upregulated dsRNA pattern recognition receptor sensor genes (TLR3, MDA5) involved in IFN pathway activation
	GSK-LSD1 decreased protein expression of key LSD1 activity dependent-RISC complex components (DICER, AGO2, TRBP2) involved in IFN/ISG activation
	GSK-LSD1 increased lysine 726 (K726me1) monomethylation status on AGO2 is associated with AGO2 instability
	LSD1 expression level is inversely correlated with IFN response in BRCA TCGA patients
Qin et al. [95]	LSD1 is negatively correlated with CD8+ T cell attracting chemokines (CCL5, CXCL9, CXCL10) and PD-L1 in aggressive TNBC or ER(−) breast cancer
	LSD1 mRNA expression is elevated in ER(−) or basal-like breast cancer
	LSD1 inhibitors (HCI-2509, Tranylcypromine/TCP) increased expression of PD-L1, CCL5, CXCL9, CXCL10 in MDA-MB-231 cells
	Increased H3K4me2 enrichment at proximal elements or core regions of transcription start sites at promoters of chemokines and PD-L1
	HCI-2509 dose-dependently increased PD-L1 and T cell chemokine expression in human (MDA-MB-231) and murine (4T1, EMT6) TNBC lines
	HCI-2509 promoted upregulation of PD-L1 basal-surface expression in TNBC cells
	HCI-2509 increased the migration of CD8+ T cells
	HCI-2509 decreased mRNA expression of PD-1 and reduced cell surface expression of PD-1 in activated CD8+ cells
	HCI-2509 coupled PD-1 mAb increased mRNA expression of PD-L1, CCL5, CXCL9, CXCL10 and CCR5 in EMT6 tumors
	HCI-2509 coupled PD-1 mAb increased CD8+ T lymphocyte recruitment and CD3+ CD8+ T cells in lymph nodes adjacent to EMT6 tumors
	HCI-2509 coupled-PD-1 mAb attenuated CD4+/CD8+ ratio in lymph nodes adjacent to EMT6/4T1 tumors
Condamine et al. [97]	FAD-directed covalent inhibitor of LSD1 (INCB059872) oral administration decreased PMN-MDSC population and increased macrophage population in 4T1 cancer model
	INCB059872 increased intratumoral T lymphocyte infiltration
	INCB059872 coupled anti-PD-L1 antibody enhanced antitumor efficacy in 4T1 cancer model
Ji et al. [101]	LSD1 inhibitor (GSK-LSD1) and 5-FU chemotherapy increased CD8+ T cell infiltration and improved tumor progression in T-IC enriched cells from 4T1-chemoresistant model
	GSK-LSD1 increased demethylated H3K4 status, increased differentiation-related genes (FOXA2, HNF4A) and decreased stemness-maintenance markers (Oct-4, Sox-2)
	GSK-LSD1 induced accumulation of ERV transcripts (IAP, MusD, LINE-1), upregulated levels of MDA5 and IFN-β in chemoresistant-4T1 cells
	GSK-LSD1 alone or GSK-LSD1 coupled 5-FU downregulated T-IC population and enhanced T-cell mediated antitumor immunity (increased CD8+ T cell infiltration, increased CD8+/Treg ratio) in chemoresistant-4T1 cells
	GSK-LSD1 alone or GSK-LSD1 coupled 5-FU reduced postsurgical relapse rates and metastatic capacities in chemoresistant-4T1 cells
	GSK-LSD1 coupled paclitaxel/gemcitabine similarly reduced T-IC population and increased CD8+ T cell recruitment in 5FU-induced chemoresistant TNBC
	GSK-LSD1 coupled epirubicin further reduced Sox-2 high cell population and increased tumor-infiltrating CD8+ T cells in 5FU-induced chemoresistant TNBC
Tu et al. [38]	Nuclear serine 111 phosphorylated-LSD1 (nLSD1p) expression is enriched in chemotherapy-resistant CTC, MDA-MB-231 and TNBC xenografts
	LSD1 inhibitors (dual FAD/CoREST-targeting “phenelzine”; reversible cell-permeable peptidomimetic “EPI-111”) decreased nuclear LSD1 expression and increased nuclear H3K4me2 in MDA-MB-231 cells
	Phenelzine and EPI-111 reduced EMT markers (CSV, ABCB5) in 4T1 and MDA-MB-231 cells
	Triple-therapy (phenelzine coupled anti-PD-1 and abraxane) or bi-therapy (phenelzine coupled anti-PD-1 or abraxane) further reduced tumor burden, mesenchymal (CSV, LSD1p, ALHD1A) and stem-like (CD133, ALDH1A, ABCB5) markers in 4T1 model
	Phenelzine induced all immune function-related pathways (T cell function, cytokines and receptors, interleukins, and CD molecules), increased expression of CD8+ T cell-related immune effect genes, and dramatically increased IFN-γ gene expression in 4T1 model
	Phenelzine alone or in combination exhibited superior induction in CD8+ T cell infiltration, particularly CD8+ IFN-γ+ T cell infiltration in 4T1 model
	EPI-111 induced overexpression of all immune-related pathways in 4T1 model
	EPI-111 increased CD45+ and CD3+ CD45+ T cell infiltration, increased CD8+ T cell infiltration, reduced CD45− population and checkpoint markers for exhausted CD8+ T cells in 4T1 model
	Phenelzine or EPI-111 monotherapy increased CD8+ T cells and total IFN-γ+ CD8+ T cell infiltration (compared to anti-PD-1 or abraxane monotherapy) in 4T1 model
	Phenelzine increased Tem population (CD45RA− CCR7−; effector memory) from 20.1 to 24.2%, Temra population (CD45RA+ CCR7−; effector memory re-expressing) from 33.5 to 34%, total CD8+ IFN-γ expression from 49 to 50%, Ki67 and IFN-γ protein expression in a stage IV metastatic breast cancer patient treated with paclitaxel/trastuzumab/pertuzumab

**Table 2:** Completed clinical trials on LSD1 inhibitors for breast cancers.

Study Phase	Drug	Malignancy	Clinical Trials.Gov Identifier	Study Description	Clinical Features
Open-label phase I (EPI-PRIMED study) [110]	Phenelzine (coupled nab-paclitaxel)	Locally advanced (inoperable) or metastatic breast cancer	NCT03505528	Double-agent	Phenelzine treatment decreased cytoplasmic expression of all mesenchymal markers (CSV, EGFR, FOXQ1, PD-L1, SNAI1) and cytoplasmic LSD1 expression
				Eight patients (*n* = 8) with at least one dose of phenelzine and nab-paclitaxel	Dose-dependent inhibition in phosphorylated LSD1 (LSD1p) nuclear expression
				Cohort median age of 59 years (range 35–73)	Enhanced H3K4me2 and H3K9me2 nuclear signal
				Three triple-negative patients (*n* = 3), five estrogen-receptor positive patients (*n* = 5)	Time-dependent reduction in PD-L1 expression
				No treatment-related deaths and no life-threatening events	
				4/8 of patients were alive with median PFS of 34 weeks, after median follow-up of 113 weeks	
				2 TNBC patients had survival rate (57, 76 weeks), while 1 TNBC patient was still alive (149 weeks)	
